# Henipavirus RNA in African Bats

**DOI:** 10.1371/journal.pone.0006367

**Published:** 2009-07-28

**Authors:** Jan Felix Drexler, Victor Max Corman, Florian Gloza-Rausch, Antje Seebens, Augustina Annan, Anne Ipsen, Thomas Kruppa, Marcel A. Müller, Elisabeth K. V. Kalko, Yaw Adu-Sarkodie, Samuel Oppong, Christian Drosten

**Affiliations:** 1 Bernhard Nocht Institute for Tropical Medicine, Hamburg, Germany; 2 Institute of Virology, University of Bonn Medical Centre, Bonn, Germany; 3 Noctalis, Centre for Bat Protection and Information, Bad Segeberg, Germany; 4 Kumasi Centre for Collaborative Research in Tropical Medicine (KCCR), Kumasi, Ghana; 5 Institute of Experimental Ecology, University of Ulm, Ulm, Germany; 6 Smithsonian Tropical Research Institute, Balboa, Panama; 7 Kwame Nkrumah University of Science and Technology, Kumasi, Ghana; University of Pretoria, South Africa

## Abstract

**Background:**

Henipaviruses (Hendra and Nipah virus) are highly pathogenic members of the family *Paramyxoviridae*. Fruit-eating bats of the Pteropus genus have been suggested as their natural reservoir. Human Henipavirus infections have been reported in a region extending from Australia via Malaysia into Bangladesh, compatible with the geographic range of Pteropus. These bats do not occur in continental Africa, but a whole range of other fruit bats is encountered. One of the most abundant is *Eidolon helvum*, the African Straw-coloured fruit bat.

**Methodology/Principal Findings:**

Feces from *E. helvum* roosting in an urban setting in Kumasi/Ghana were tested for Henipavirus RNA. Sequences of three novel viruses in phylogenetic relationship to known Henipaviruses were detected. Virus RNA concentrations in feces were low.

**Conclusions/Significance:**

The finding of novel putative Henipaviruses outside Australia and Asia contributes a significant extension of the region of potential endemicity of one of the most pathogenic virus genera known in humans.

## Introduction

The subfamily *Paramyxovirinae* in the family *Paramyxoviridae* comprises the five genera Respiro-, Morbilli-, Rubula-, Avula-, and Henipavirus, as well as a group of yet unclassified viruses [1]. The genus Henipavirus contains two of the most pathogenic viruses known in humans, Hendra- and Nipah virus, which were discovered only in 1994 and 1998, respectively [2]–[5]. Both viruses cause severe encephalitis in humans, exemplified by the Nipah virus outbreaks in Malaysia, India and Bangladesh with case fatality rates ranging from 40–100% [6]–[10]. Work on these viruses is restricted to biosafety level-four laboratories. Typically, Henipaviruses are not circulating in humans, but can be acquired from domestic animals such as pigs or horses [6], [11]. Direct transmission of Nipah virus between humans has been described and transmission from bats to humans was implicated in Nipah virus outbreaks not related to pig farming [12]–[14]. Drastic control measures may be necessary to contain outbreaks of Henipaviruses, as exemplified by the culling of more than one million pigs during a Nipah virus outbreak in Malaysia in 1999 [11]. Henipaviruses seem to have their primary reservoir in fruit bats of the genus Pteropus, in the family Pteropodidae. Serologic evidence for Henipavirus infection in bats has been reported in a geographic range covering Australia, Malaysia, Thailand, Cambodia, Indonesia, Bangladesh, India, and Madagascar [9], [15]–[20]. Hendra virus was isolated from bats in Australia, and Nipah virus in Malaysia and Cambodia. Detection of Henipavirus RNA has been accomplished in Thailand (Nipah virus) and Australia (Hendra virus) [20]. Consequently, the geographic range of Pteropus seems to limit the geographic range of Hendra and Nipah virus encephalitis in humans [8], [20], [21].

Interestingly, a recent serological study on *E. helvum*, a fruit-eating bat that occurs in Africa but not in Asia, implicated the presence of antigenetically related viruses [22]. This is remarkable because *E. helvum* is highly abundant in Africa and colonies are believed to conduct annual transcontinental migration, following the rainfall gradient to suitable feeding grounds, with reports on individual bats travelling up to 2,000 kilometres [23], [24]. *E. helvum* is a rather large fruit bat that roosts exposed and at high densities in trees during daytime. The animals are frequently hunted and consumed by humans as a supplementary source of protein [25]–[27].

In spite of serological evidence, it has never been confirmed so far that Henipaviruses exist in African bats. In the current study we positively identified putative Henipaviruses for the first time in *E. helvum* in Ghana. Jointly with recent serological data [22], our results suggest a tremendous extension of the geographic range of this virus genus, which is one of the most pathogenic known to humans.

## Materials and Methods

For all capturing and sampling, permission was obtained from the Wildlife Division, Forestry Commission, Accra, Ghana. Samples were exported under a state contract between the Republic of Ghana and the Federal Republic of Germany, and under an additional export permission from the Veterinary Services of the Ghana Ministry of Food and Agriculture.

Geographic co-ordinates of the sampling site were N06°42′02.0″ W001°37′29.9″. Bats were identified as *E. helvum* by trained field biologists on site. Additionally, of those samples that yielded positive RT-PCR results, mitochondrial DNA was amplified and sequenced for species confirmation as described [28]. Bat droppings were collected on plastic film. Ca. 100 mg of feces were suspended in 500 µl of RNAlater solution immediately after dropping. Suspensions were homogenised by vortexing and 50 µl were suspended into 560 µl of Buffer AVL from the Qiagen viral RNA mini kit (Qiagen, Hilden, Germany) and processed further according to the instructions of the manufacturer as described earlier [29]. Elution volume was 50 µl. Broad-range PCR for the genus Henipavirus was done as recently described, yielding amplicons of 496 base pairs located across domains I and II of the polymerase gene [30]. Sanger sequencing of PCR products was done using dye terminator chemistry (Applied Biosystems). Bayesian inference of phylogeny was done using BEAST, version 1.4.8 [31] with both nucleotide- (GTR+I+gamma) and codon-based (SRD06) substitution models [32]. The most applicable population model was determined by using both the ESS statistic for all traces and the Bayes factor test on the posterior probability trace of each run with TRACER. Metropolis-coupled Markov-chain Monte Carlo (MCMC) chains with 2X10E7 iterations were sampled every 1000 generations, resulting in 20,000 sampled trees.

Based on prior findings [33], [34], isolation of virus was attempted from feces suspended in RNAlater solution. Vero and CaCo2 cells were used, as well as primary cells from colon, lung and kidney of *Myotis nattereri*, *E. helvum* and *Rousettus aegyptiacus* bats (own unpublished data). No cytopathic effect was observed and no virus growth was seen by RT-PCR, despite repeated trials (data not shown).

## Results

**Figure 1:**
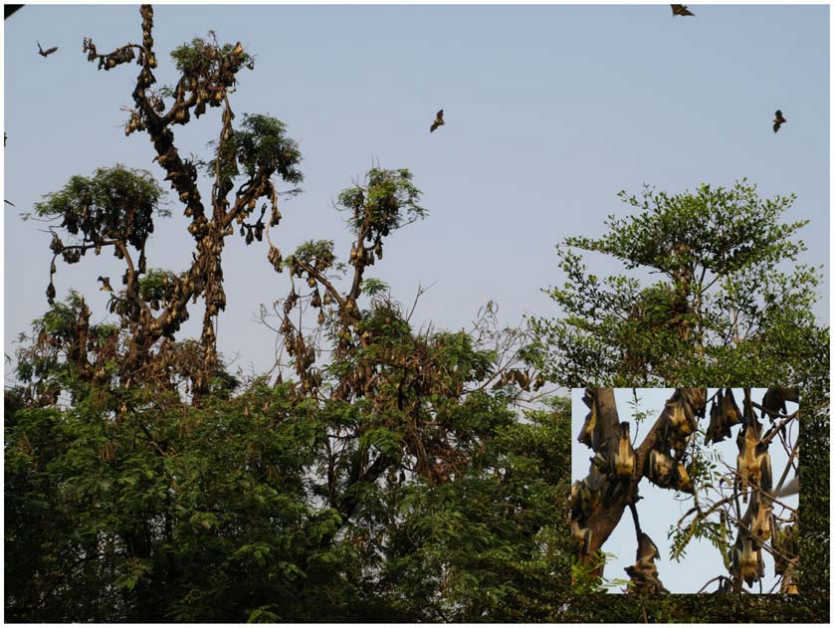
The *Eidolon helvum* colony at Kumasi zoological gardens, Ghana. Photos from A. Seebens and F. Gloza-Rausch.

During February 2008, a large colony of *E. helvum* fruit bats was studied in the zoological gardens of Kumasi, Ghana (Figure 1). The colony was estimated to contain approximately 400,000 bats [35]. During five days in early February, plastic film (12 m^2^) was laid out in the late afternoon and early in the morning under several trees densely occupied by bats, in order to collect fecal samples. Due to a low frequency of droppings received on the plastic film (about one dropping per 2 minutes), and due to the distance between the individual droppings on plastic film, it was assumed that individual droppings stemmed from different individual bats.

**Figure 2:**
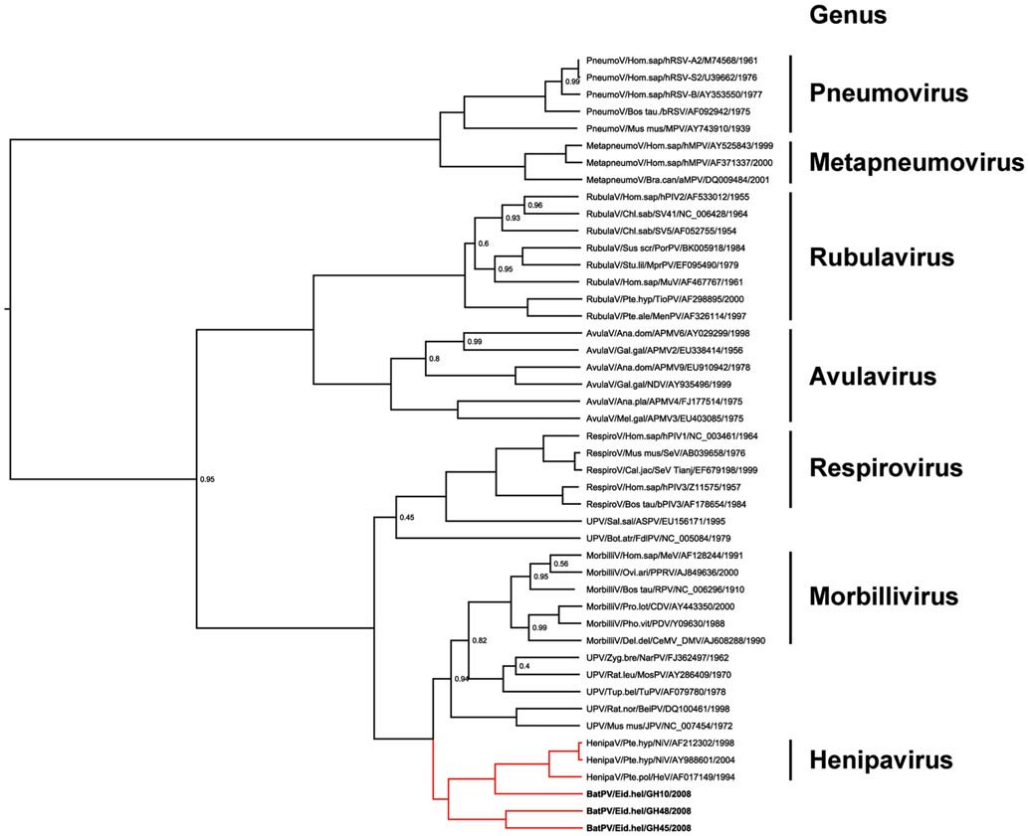
L-gene phylogeny with novel bat paramyxoviruses. The phylogenetic tree was generated and annotated using BEAST version 1.4.8, using a codon-based substitution and an expansion growth population model [31]. For better graphic visualization, only posterior probability values below 1.0 are shown. The initial sequence alignment was based on a 496 base pair fragment from PCR screening. Exclusion of primer sites resulted in a 439 bp fragment, and further exclusion of ambiguous sites resulted in a 426 base pairs gap-free alignment corresponding to nucleotides 9993–10430 of Human parainfluenza virus 1 strain Washington/1964, GenBank Accession Number NC_003461. Novel bat paramyxoviruses are shown in boldface type. GenBank Accession numbers of the novel viruses are FJ609191 (BatPV/Eid.hel/GH10/2008), FJ609194 (BatPV/Eid.hel/GH48/2008) and GQ168929 (BatPV/Eid.hel/GH45/2008). Branches leading to the Henipavirus genus are in red color. Established genera in the *Paramyxoviridae* family are indicated next to taxon names. Taxa are named according to the following pattern: Genus/typical host/virus abbreviation/accession number/isolation year. HenipaV = Henipavirus, MorbilliV = Morbillivirus, RespiroV = Respirovirus, RubulaV = Rubulavirus, AvulaV = Avulavirus, MetapneumoV = Metapneumovirus, PneumoV = Pneumovirus, UPV = unclassified paramyxovirus. NiV = Nipah virus, HeV = Hendra virus, PPRV = Peste-des-petitsruminants virus, CeMV DMV = Cetacean morbillivirus strain dolphin morbillivirus, MeV = Measles virus, RPV = Rinderpest virus, PDV = Phocine distemper virus, CDV = Canine distemper virus, SeV = Sendai virus, bPIV3 = Bovine parainfluenza virus 3, hPIV1 = Human parainfluenza virus 1, hPIV3 = Human parainfluenza virus 3, SV5 = Simian parainfluenza virus 5, SV41 = Simian virus 41, MenPV = Menangle virus, MprPV = Mapuera virus, MuV = Mumps virus, PorPV = Porcine rubulavirus, TioPV = Tioman virus, hPIV2 = Human parainfluenza virus 2, aMPV4 = Avian paramyxovirus type 4, aMPV6 = Avian paramyxovirus type 6, aMPV9 = Avian paramyxovirus type 9, aMPV2 = Avian paramyxovirus type 2, aMPV3 = Avian paramyxovirus type 3, NDV = Newcastle disease virus, hMPV = Human metapneumovirus, aMPV = Avian metapneumovirus, MPV = Murine pneumonia virus, bRSV = Bovine respiratory syncytial virus, hRSV = Human respiratory syncytial virus, NarPV = Nariva virus, ASPV = Atlantic salmon paramyxovirus, TuPV = Tupaia paramyxovirus, MosPV = Mossman virus, BeiPV = Beilong virus, JPV = J virus, FdlPV = Fer-de-lance virus.

In total, three of 215 fecal samples yielded RT-PCR products. 496 base pair fragments of all products were sequenced and aligned with homologous fragments of *Paramyxoviridae* reference strains, including Hendra and Nipah viruses from GenBank. Supplemental figures S1 and S2 show nucleotide and amino acid alignments of the novel bat paramyxoviruses with Hendra and Nipah virus prototype strains. Refer to Figure 2 for GenBank Accession Numbers of all reference strains, including those of the novel viruses described here.

As shown in Figure 2, the resulting phylogeny was in concordance with current proposals of *Paramyxoviridae* taxonomy. The three viral sequences from *E. helvum* were in close or basal association with the currently established genus Henipavirus (Figure 2). One virus was most closely related to Nipah viruses, extending the internal amino acid distance within the genus Henipavirus from 6.8% to 32.9% (Table 1). Inclusion of the two other viruses extended the internal distance to 36.3%. In the analyzed fragment, the highest internal distance in any mammalian paramyxovirus genus was observed in the genus Rubulavirus at 40.8%, justifying association of the novel bat paramyxoviruses with the genus Henipavirus.

**Table 1:** Percentage amino acid identity.

	Genus/Species	[1]	[2]	[3]	[4]	[5]	[6]	[7]	[8]	[9]	[10]	[11]	[12]
[1]	**Pneumovirus**	≥53.8											
[2]	**Metapneumo-virus**	49.4–55.8	≥82.1										
[3]	**Avulavirus**	18.9–26.4	21.6–30.4	≥40.7									
[4]	**Rubulavirus**	19.3–26.7	22.0–27.3	37.3–50.7	≥59.2								
[5]	**Respirovirus**	19.4–25.7	22.9–25.7	27.8–38.2	35.6–43.8	≥69.9							
[6]	**Morbillivirus**	21.5–26.4	27.1–31.2	29.9–35.4	34.2–42.5	50.0–56.2	≥77.4						
[7]	**Hendra virus** a	20.1–22.9	26.4–27.8	29.9–36.1	38.4–43.8	50.7–53.4	60.3–64.4	100					
[8]	**Nipah virus Bangladesh** b	20.1–23.6	26.4–27.8	31.2–35.4	40.4–43.8	51.4–54.1	62.3–65.1	93.2	100				
[9]	**Nipah virus Malaysia** c	20.1–23.6	26.4–27.8	31.2–34.7	39.7–43.2	52.1–54.1	62.3–65.1	93.8	99.3	100			
[10]	**BatPV/Eid.hel/GH10/2008**	23.6–24.3	25.7–25.7	30.6–35.4	37.7–43.2	50.0–50.0	61.6–64.4	65.8	67.1	67.1	100		
[11]	**BatPV/Eid.hel/GH45/2008**	23.6–25.7	25.7–27.1	29.2–32.6	36.3–41.1	51.4–54.8	58.2–64.4	61.6	63.7	63.7	58.2	100	
[12]	**BatPV/Eid.hel/GH48/2008**	25.0–26.4	27.1–28.5	27.8–34.0	35.6–40.4	52.7–54.1	57.5–63.0	61.6	63.7	63.0	61.6	69.9	100

Amino acid identity from analysis between sequences is shown. All positions containing alignment gaps and missing data were eliminated only in pairwise sequence comparisons (Pairwise deletion option). There were a total of 159 positions in the final dataset. Located across domains I–II of the viral RdRp (nucleotides 9993–10430 in Human parainfluenza virus 1 strain Washington/1964, NC_003461). Highest identity of bat paramyxoviruses with established species in boldface type.

a AF017149.

b AY988601.

c AF212302.

After sequencing, individual real time PCR assays with TaqMan probes were designed for each paramyxovirus sequence (assay formulations available on request). Individual *in-vitro* transcribed RNA standards were generated for each sequence with methodology described earlier [36], and the paramyxovirus RNA concentrations in samples were determined. The sample that yielded strain BatPV/Eid.hel/GH10/2008 contained 1,030 RNA copies per milligram of feces. The two other samples (BatPV/Eid.hel/GH45/2008; BatPV/Eid.hel/GH48/2008) contained below 50 copies of RNA per milligram, i.e., they were detected but ranged below the limit down to which reliable quantification was possible.

## Discussion

Further to serological data provided by Hayman et al. [22], we have detected paramyxoviruses closely related to Hendra and Nipah virus in an African fruit bat, *E. helvum*. The genetic distance between these viruses and Hendra and Nipah virus was compatible with the distance observed within other established *Paramyxoviridae* genera, suggesting that the novel viruses might constitute members of the Henipavirus genus. The considerable sequence divergence between the three novel putative Henipaviruses encountered in one huge colony might indicate substantial genetic variability of related viruses in bats. Formal taxonomic classification according to conventions for *Paramyxoviridae* will require the characterization of additional genomic features, such as the phylogenies of the nucleoprotein and F genes, the lengths of intergenic regions, as well as the predicted transcription start sites of subgenomic RNAs [1], [3]. Unfortunately, sequencing of longer genomic fragments was not successful from our samples, most likely due to the low concentration of RNA encountered. This was also the assumed reason for failure of virus isolation.

The novel viruses were detected from wild *E. helvum* that occupied trees in a zoological garden in the centre of Kumasi, Ghana's second largest city with a population of 1.5 million. Large colonies of *E. helvum* are widely observed in urban areas of Subsaharan Africa [27], [37]. In the location studied here, hundreds of visitors and staff enter the zoo on a daily basis and may become exposed. The ways of exposure may be key features in the understanding of the origin of epidemics of bat-borne viruses, such as Ebolaviruses, Henipaviruses, or Coronaviruses [9], [38]–[41]. It has been proposed that humans may be exposed to viruses from fruit bats that chew fruits and spit out the pulp at feeding sites [42]. However, it appears that *E. helvum* uses urban habitats only for roosting and rarely for feeding. Nevertheless, detailed studies on the foraging behavior of *E. helvum* are lacking. Another way of exposition may be via contact with bat urine or fecal material. In this study we have sampled bat feces that are abundant under trees in urban *E. helvum* roosting sites. Interestingly, the observed virus RNA concentrations in fecal material were rather low as compared to enteric viruses transmitted via the fecal-oral route in humans [43]–[45]. Bat urine was frequently described to contain Henipaviruses [15], [41], [46]. Despite immediate recollection of fecal samples from plastic sheets in this study, contamination of these samples with bat urine cannot be completely excluded. Overall, our data suggests a limited risk of exposure to virus from bat feces. This is important to note to prevent any premature action aiming at extirpating flying foxes as potential virus hosts, as this may disrupt important ecological functions, i.e., seed dispersal and pollination. However, our sample size was limited and more studies are required to determine whether RNA concentrations may vary within bat colonies, and over time.

Another way of exposure to bat viruses may be via consumption of bat meat for food or medical purposes [25], [26]. *E. helvum* is known to be one of the preferred bat species hunted for wild game in Africa [47]. Future studies need to focus on whether there is a relevant concentration of virus in organs or meat of bats, and whether persons consuming bat meat on a regular basis might show evidence of past infection.

## Supporting Information

Figure S1 Nucleotide alignment of Henipavirus reference strains and novel viruses. The entire 439 base pair polymerase fragment is aligned with Hendra virus and Nipah virus reference strains. Hendra virus on top serves as the comparison sequence in the alignment. Dots represent identical bases in compared sequences; deviations are spelled out.
(0.02 MB PDF)

Figure S2 Amino acid alignment of Henipavirus reference strains and novel viruses. The 146 amino acids resulting from translation of a 439 base pair polymerase fragment are aligned with Hendra virus and Nipah virus reference strains. Hendra virus on top serves as the comparison sequence in the alignment. Dots represent identical amino acids in compared sequences; deviations are spelled out.
(0.01 MB PDF)
