# Supplementary material for: Henipavirus RNA in African Bats
Source: PLoS One. 2009 Jul 28;4(7):e6367. doi: 10.1371/journal.pone.0006367 (PMC2712088; doi:10.1371/journal.pone.0006367)
Supplement: Figure S1 — Nucleotide alignment of Henipavirus reference strains and novel viruses. The entire 439 base pair polymerase fragment is aligned with Hendra virus and Nipah virus reference strains. Hendra virus on top serves as the comparison sequence in the alignment. Dots represent identical bases in compared sequences; deviations are spelled out. (0.02 MB PDF) [file pone.0006367.s001.pdf]

```
                                     10        20        30        40        50        60        70        80
                             ....|....|....|....|....|....|....|....|....|....|....|....|....|....|....|....|
Hendra virus AF017149        GTACCGTGACAGGCATCGGGGAGCTTGGCCTCCACTTTATCTGCCATCGCATGCATCGAAACACATAATAAGACTCAAGA
Nipah virus AY988601         ...TA.G..T..A..CG.A..G..C........T..C..C..C..CG.A........T..G..T.....CC.CT.G..A.
Nipah virus AF212302         ...TA.G..T..A..CG.A.....C........T.....C..C..CG.A........T.....T.....CC.TT.G..A.
BatPV/Eid.hel/GH10 FJ609191  T........T.AA...G.A..G.T.........T..AACCT.T...AA.....T.CATC.GG.A..C.A......AC.AT
BatPV/Eid.hel/GH45 GQ168929  ...TA.A..T..A...G....TA.......A..CTG.A....T..TC.C...TGT..ACCTGC...TCG..A.AAAC.AC
BatPV/Eid.hel/GH48 FJ609194  A.T...G..T..A...G.A..T.TG.....CT.TTG..TC..T..TGAT.......TCC.TTA..C.A.TCCAAA....

                                     90       100       110       120       130       140       150       160
                             ....|....|....|....|....|....|....|....|....|....|....|....|....|....|....|....|
Hendra virus AF017149        ATTCTGGTGAATCATTAACAGTTGATGATTGTGTTAAGAACTGGGAGTCATTTTGTGGGATTCAATTTGATTGCTTTATG
Nipah virus AY988601         .......G.....T.....CA.............C...........A.....C....................T..C...
Nipah virus AF212302         .......G.....T..G..CA.......C.....C.....T.....A.....C....................T..C...
BatPV/Eid.hel/GH10 FJ609191  CA..A..G...AG.C.G..TTA...AATA...A....AC.....A.A........C...C.GA.T...A.A..T......
BatPV/Eid.hel/GH45 GQ168929  TAAA..CA......G.GT..A.A..G..C...A..C....T...C.A...........AT.CAGT..CA.G..T......
BatPV/Eid.hel/GH48 FJ609194  TGAAC..A..GG....G...A.A..G.....C.CAG....T...A....G........AT..A.C...........C...

                                    170       180       190       200       210       220       230       240
                             ....|....|....|....|....|....|....|....|....|....|....|....|....|....|....|....|
Hendra virus AF017149        GAGTTAAAATTAGACAGTGATTTGAGCATGTATATGAAAGATAAAGCACTATCCCCAATAAAGGAGGAATGGGATAGTGT
Nipah virus AY988601         ...C.G....................T....................CT.G..T..T..C..A..C..G.....C.....
Nipah virus AF212302         ...C.G.....G.........C....T....................TT....T.....C..A..C........C.....
BatPV/Eid.hel/GH10 FJ609191  ..TC.T..T..G..TGAA..C.................G........T..TG.TG.G....G.A.T..G.....C.....
BatPV/Eid.hel/GH45 GQ168929  CCA...TC......TTCA...C.T..T.....C.....G..C......T....A..T.AT..ATCA........CTC...
BatPV/Eid.hel/GH48 FJ609194  CCC..G.C.C....TGA...CC....T.....C.....G........TT....G.....TCGATCAC........TCA..

                                    250       260       270       280       290       300       310       320
                             ....|....|....|....|....|....|....|....|....|....|....|....|....|....|....|....|
Hendra virus AF017149        CTATCCACGTGAAGTACTTAATTACACGCCACCCAGATCTACCGAACCAAGAAGATTGGTGGATGTATTTGTCAATGATG
Nipah virus AY988601         A........A.....G..G.GC..T..C.....G.A...A.....G..............T..C..T.....A..C....
Nipah virus AF212302         A..C...........GT.G.GC..T..C.....G.AG..A.....G..............T..C..T.....A.......
BatPV/Eid.hel/GH10 FJ609191  T.....TAAG...AAT..CTC....CA....A.A.A.AA...AACT..T..G..G..A......TAC...A.........
BatPV/Eid.hel/GH45 GQ168929  ......CGCAAC.AACACC..A.....A.....T......CAAAC.T.T..G.....A..T...AGG..CA.TG.A....
BatPV/Eid.hel/GH48 FJ609194  A..C..TACATCCAATA.G.CC....AT..T...T.TT.G..ATC.T.T........A..T........CA.........

                                    330       340       350       360       370       380       390       400
                             ....|....|....|....|....|....|....|....|....|....|....|....|....|....|....|....|
Hendra virus AF017149        AGAATTTCGATCCTTATAATATGTTAGAGTATGTCCTTACAGGAGATTATTTAACTGATGAACAATTCAATGTTTCTTAT
Nipah virus AY988601         .A..C..T.....G..C..C...C....A......T.GT.C.C..C....C.CGAA.....T.................C
Nipah virus AF212302         .A..C..T.....A..C..C...C.G..A......T.AT.C.T..C....C.CGAG........................
BatPV/Eid.hel/GH10 FJ609191  .AG...............GC...C.CC.......GA.A.AC..T......C.G.A...CCTG..T........A..G...
BatPV/Eid.hel/GH45 GQ168929  .TG.G..T..A..A.C.G.C...A.CA.T.........T....T..G...C.C.G......TAC...T...A.C..A..C
BatPV/Eid.hel/GH48 FJ609194  CTG.C..T..A....CG..AT.AA.T..T......A.C.GC.....A..C...GA...CA..G........A.C..C...

                                    410       420       430
                             ....|....|....|....|....|....|....|....
Hendra virus AF017149        AGTCTAAAGGAGAAAGAAACTAAACAAGCAGGAAGATTA
Nipah virus AY988601         ...T.G...........G..A..G..G..T...C....G
Nipah virus AF212302         ..CT.G...........G..G..G.....T...C....G
BatPV/Eid.hel/GH10 FJ609191  ........A........G..A..GG...T.......C.C
BatPV/Eid.hel/GH45 GQ168929  ........A..A......GTC...G...TC...C.....
BatPV/Eid.hel/GH48 FJ609194  .....C..A..A.....GGTG...G.G.TG..TC.TC.T
```
