# Supplementary material for: Henipavirus RNA in African Bats
Source: PLoS One. 2009 Jul 28;4(7):e6367. doi: 10.1371/journal.pone.0006367 (PMC2712088; doi:10.1371/journal.pone.0006367)
Supplement: Figure S2 — Amino acid alignment of Henipavirus reference strains and novel viruses. The 146 amino acids resulting from translation of a 439 base pair polymerase fragment are aligned with Hendra virus and Nipah virus reference strains. Hendra virus on top serves as the comparison sequence in the alignment. Dots represent identical amino acids in compared sequences; deviations are spelled out. (0.01 MB PDF) [file pone.0006367.s002.pdf]

```
                                     10        20        30        40        50        60        70        80
                             ....|....|....|....|....|....|....|....|....|....|....|....|....|....|....|....|
Hendra virus AF017149        YRDRHRGAWPPLYLPSHASKHIIRLKNSGESLTVDDCVKNWESFCGIQFDCFMELKLDSDLSMYMKDKALSPIKEEWDSV
Nipah virus AY988601         .....G.........A.................I........................................D.....
Nipah virus AF212302         .....G.........A.................I........................................D.....
BatPV/Eid.hel/GH10 FJ609191  ...K.G.V....TF.K.VHQE.K..QS...R..YEI.I.H.K....LN.K...D.N..E............AA.RN.....
BatPV/Eid.hel/GH45 GQ168929  .....G.T...CN..P.C.PA.RKKQLNA..VSIE..IQ..Q....FS.K...P.S................N.S.....
BatPV/Eid.hel/GH48 FJ609194  F....G.V..SCF..D...PL.KSK.MN..A..IE..AE..K....FN.....P.T..D..............RSQ....

                                     90       100       110       120       130       140
                             ....|....|....|....|....|....|....|....|....|....|....|....|....|.
Hendra virus AF017149        YPREVLNYTPPRSTEPRRLVDVFVNDENFDPYNMLEYVLTGDYLTDEQFNVSYSLKEKETKQAGRL
Nipah virus AY988601         ......S....K...........................S.A..E.D...................
Nipah virus AF212302         ......S....K...........................S.A..E.....................
BatPV/Eid.hel/GH10 FJ609191  ..K.N.S.Q.TKN.T......Y.I...D....S..Q..IN....N.PD.............EV...
BatPV/Eid.hel/GH45 GQ168929  ..ATNTK......QTS.....R.IE.DE.E.SD.IN...S.E..S.DT..I........V.EV...
BatPV/Eid.hel/GH48 FJ609194  ..TSNMT.N..I..SS.......I..AD.E.SKLID..IS.E..D.KE..I........V.EV...
```
